# Towards a physically more active lifestyle based on one’s own values: study design of a randomized controlled trial for physically inactive adults

**DOI:** 10.1186/1471-2458-13-671

**Published:** 2013-07-19

**Authors:** Anu Maarit Kangasniemi, Raimo Lappalainen, Anna Kankaanpää, Janne Kulmala, Harto Hakonen, Tuija Tammelin

**Affiliations:** 1 LIKES - Research Center for Sport and Health Sciences, Jyväskylä, Finland, Viitaniementie 15 a, Jyväskylä 40720, Finland; 2 Department of Psychology, University of Jyväskylä, Jyväskylä, Finland

**Keywords:** Acceptance and Commitment Therapy, ACT, Physical activity, Behaviour, Effectiveness, Adults, Psychological well-being

## Abstract

**Background:**

This randomised controlled trial demonstrates the effectiveness of a value-based intervention program to encourage a physically more active lifestyle among physically inactive adults aged 30 to 50 years. The conceptual framework of the program is based on an innovative behavioural therapy called Acceptance and Commitment Therapy (ACT) that aims to increase an individual’s psychological flexibility and support behaviour change towards a higher quality and more meaningful life.

**Methods:**

Participants will be randomly allocated to a feedback group (FB) or an Acceptance and Commitment based (ACT + FB) group. Both the groups will receive written feedback about their objectively measured physical activity levels and offered an opportunity to attend a body composition analysis. In addition, the Acceptance and Commitment based group will attend six group sessions and be given a pedometer for self-monitoring of their daily physical activity throughout the 9-week intervention. The group sessions aim to clarify individual values and enhance committed actions towards the goal of achieving a more meaningful life. Participants will also be taught new skills to work on subjective barriers related to physical activity. Physical activity will be measured objectively by an accelerometer over seven consecutive days and by self-reported questionnaires at the baseline, as well as at 3, 6, 9 and 15 months after the baseline measures. In addition, psychological well-being will be measured through the questionnaires, which assess mindfulness skills, psychological flexibility, psychological distress and depressive symptoms.

**Discussion:**

This study’s objective is to demonstrate a research protocol for a randomized controlled study motivating a physically more active lifestyle based on one’s own values among physically inactive adults. The aim of the study is to evaluate the feasibility and intervention efficacy on physical activity and psychological well-being, and discuss challenges in motivating physically inactive adults towards physically more active lifestyles.

**Trial registration:**

ClinicalTrials.gov, number NCT01796990.

## Background

The promotion of physical activity is one of the major challenges for public health. Sedentary lifestyle has become a widespread health problem and it is one of the major causes of chronic diseases, like type II diabetes and cardiovascular disease [1]. By increasing physical activity especially among physically inactive individuals, their health could be substantially improved [2]. However, despite the fact that individuals are very well aware of the health benefits of physical activity, they are often unable to take action or change their behaviour and adopt a healthier lifestyle [3]. Only half of the physical activity intervention studies have been successful in increasing physical activity levels [4]. The poor effectiveness of physical activity interventions is partly due to insufficient information about the effective methods that work, especially among the physically inactive groups. In addition, there is a great need for methods to enhance individual motivation and challenge individuals to change their lifestyles in the long run in a cost-effective manner [5].

Acceptance and Commitment Therapy (ACT) is an innovative behavioural therapy approach based on functional contextualism [6]. The core analytic unit is the ongoing act in the context and the focus is on the whole event. ACT aims to increase psychological flexibility, which can be defined as the ability to be in the present moment with full awareness and openness to experiences and to take action guided by one’s values [6,7]. Instead of focusing on how one should act or behave according to the certain instructions or recommendations (e.g., for example related to a healthier lifestyle), the emphasis is on the behaviour that works in relation to value-based goals. Research evidence has shown that ACT-based approaches may help individuals to live in more flexible ways, and have more meaningful lives according to their own values, and has proven to be powerful in overcoming many kinds of mental and health-related problems [8]. In the area of health, the results have shown ACT to be successful in the treatment of numerous health conditions (e.g., chronic pain [9], type II diabetes [10], weight regain among bariatric surgery patients [11,12] and depression [13]) and associated with better psychological and quality of life scores [6].

The ACT approach can be divided into two parts. The first part includes acceptance and mindfulness processes, and the second part includes commitment and behavioural change processes. Acceptance refers to the process in which individuals can choose to experience the full range of private experiences, without having to change or defend against them. Mindfulness is a process that concentrates on the present moment as it is, not living excessively in the past or in the future. Commitment and behavioural change processes focus on value-based actions and behaviour, which lead towards a higher quality and more meaningful life. ACT has been delivered both in individual and group psychotherapy and coaching formats, and applied to a wide variety of clinical conditions and client groups [14].

In the context of physical activity promotion, only a few studies have shown that ACT could have potential in enhancing physical activity [15,16]. However, more research is needed to test the feasibility of this approach especially among the physically inactive ones. This paper will present the research protocol and methods used in the study, which aim to evaluate the effectiveness of the value-based approach in motivating physically more active lifestyles among physically inactive adults.

## Methods

### Study design

**Figure 1:**
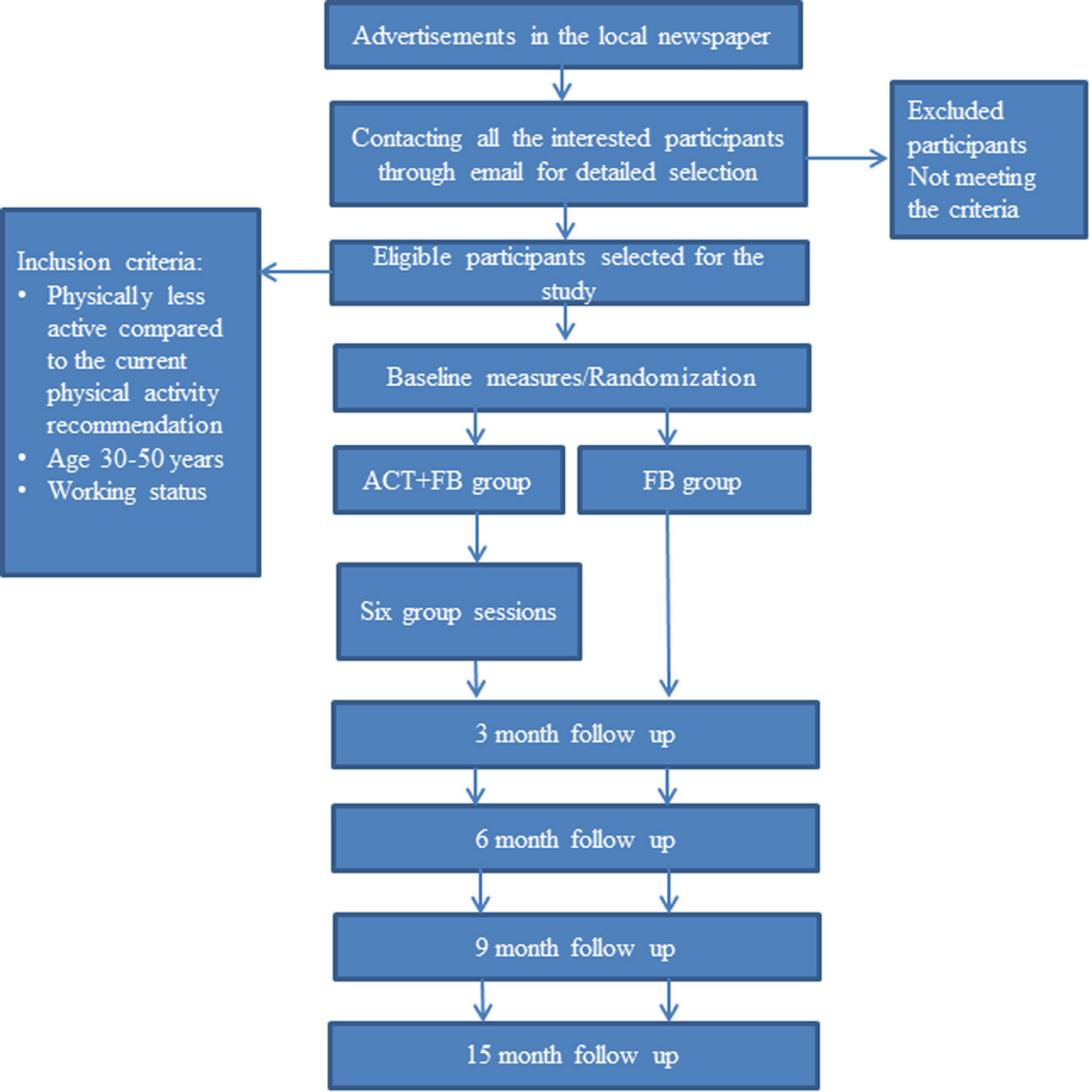
Flow diagram of the progress of the study.

The design of the study is a randomized controlled trial (Figure 1). It will evaluate the effectiveness of physical activity and psychological well-being in the Acceptance and Commitment based (ACT + FB) group versus the feedback (FB) group only.

The trial will be conducted in two phases: the first phase from autumn 2011 to December 2012, and the second phase from autumn 2012 to December 2013. The protocol was approved on 20.6.2011 by the Scientific Ethics Committee of the University of Jyväskylä, Finland. The trial is registered with ClinicalTrials.gov, number NCT01796990.

### Study population

The study population will consist of working adults aged 30 to 50 years who are physically inactive, defined as not meeting the current physical activity recommendations [1].

### Procedure for recruitment, randomization and allocation

Physically inactive adults will be recruited by advertisements in the local newspaper. All interested individuals will be screened in more detail by an online-questionnaire. The criteria for selection are: 1) age 30–50 years, 2) working status, and 3) not meeting the current physical activity recommendations [1]. The evaluation of the physical activity level will be determined by the questions: “During the last 7 days, on how many days did you carry out at least moderate intensity physical activity that lasted for at least 10 minutes each time and for a total of at least 30 minutes during one day?” The response options ranged from 0 to 7 days per week. Please include all activity for which the physical effort is moderate or harder including transportation to work and leisure time physical activity. This kind of activity accelerates the heart rate and breathing (e.g., brisk walking, running, and heavy gardening). In addition they were asked “How much time in total did you spend doing this type of physical activity during leisure time?” Estimate to the nearest half an hour [17].

All eligible participants will receive written confirmation of their acceptance into the study and be informed about the study protocol. The participants will give written informed consent if they agree to enroll in the study. Participants will be randomized into one of two parallel study groups, the Acceptance and Commitment-based group (ACT + FB) or feedback group (FB) only (see Figure 1).

### Interventions

#### Feedback group (FB)

The participants will get written feedback about their physical activity level after the baseline and after 3, 6, 9 and 15 months follow up. This individual written feedback includes information on participants’ objectively measured daily physical activity level compared to the current physical activity recommendations by using the histograms and graphs. Individual feedback will be posted home and will not include face to face interaction. As an incentive for participation, participants will also have opportunity to attend a body composition analyze and get short, personal interpretation and feedback of the results in the research center.

#### The Acceptance and Commitment-based Group (ACT + FB)

The Acceptance and Commitment-based group (ACT + FB) will be subject to the same procedures as the feedback group. In addition, they will participate in the ACT-based program and be given a pedometer for self-monitoring of their daily physical activity during the intervention program. The intervention program consists of six group sessions during the 9-week period of time, with each session lasting about 90 minutes (see Table 1). The sessions will be led by three to five group leaders, including the researcher. The group size is 8–10 participants per one group. All the group leaders will be trained in the ACT approach and supervised by the responsible researcher (AK). Participants will also get their own workbook, which will contain short descriptions of the sessions, and all individual reflections and notes.

**Table 1:** Content of the six group sessions in the Acceptance and Commitment-based group

**Topics of the group sessions**	**Key points and aims of the sessions**
1. Health behaviour	What are the factors affecting my health behaviour/well-being? What is the direction I want to move in? What are the steps I have taken in order to try to change my well-being?
2. Values and important things in life	What are the most important values for me?
	Am I living or behaving according to my values?
3. Value-based actions and barriers	What are my specific goals and actions that support my valued behaviour?
	What kind of subjective barriers or explanations have I related to physical activity?
4. Living in the present moment and self-regulation skills	How do I contact the present moment?
	How do I use mindfulness skills in order to be more aware of my own behaviour in everyday life?
5. Self-processes and physical activity	How do I see myself and how does it affect my behaviour?
	Can I be more aware of the way I am thinking about myself and learn nonreactive ways to respond to these thoughts?
6. Flexible actions	How am I doing? What are the actions which help me to achieve my desired outcomes? Do I need to change my goals? Am I living according to my values?
	Can I be more flexible in my behaviour and physically active lifestyle?

The program aims to enhance physically active lifestyles and well-being through important life values and build committed action based on the chosen important values. The program will start with an analysis of health behaviour and reflections about values and important things in life. After value clarification, participants will make concrete action plans and define their own goals. The emphasis will be placed on small and feasible everyday actions, rather than demanding exercise goals. In order to achieve the set goals, participants are taught mindfulness skills and new ways to deal with the barriers. The purpose of learning mindfulness skills is to be more aware of one’s own thoughts, emotions and automatic behaviour patterns. By noticing these automatic thinking styles or routines, participants will have a better ability to consciously change their own behaviour in the everyday life context.

Every session includes mindfulness exercises, pair and group discussions and homework related to the topic of the session. The intention of homework is to encourage participants to also work with the topics between the sessions and transfer learnt skills into their own living context. The group leader’s role is to introduce the topics, teach new skills, lead the ongoing discussion and provide a context in which participants are willing to share their own experiences, thoughts and feelings. The program does not include psycho-educational elements, counseling on health, information about the health benefits of physical activity, or concrete physical exercise.

Table 1 Content of the six group sessions in the Acceptance and Commitment Therapy (ACT) intervention group.

### Outcome measures

Measurements will take place at baseline and 3, 6, 9, and 15 months after the baseline (Figure 1). The baseline demographic characteristics of participants will also be recorded. The primary outcome measures will be based on physical activity.

### Primary outcome measures

#### Objectively measured physical activity

Physical activity will be measured objectively by an accelerometer (Actigraph GT1M, GT3X, Actigraph, Pensacola, Florida). The ActiGraph accelerometer is a small and light instrument that records integrated acceleration information as an activity count, providing an objective estimate of the intensity of vertical bodily movement. Participants are instructed to wear the accelerometer on their right hip, attached by an elastic belt during all waking hours for seven consecutive days [18]. Outcome variables were: sedentary time (< 100 counts per minute, cpm), time spent on light intensity physical activity (LPA time, min/day, 100–759 cpm), time spent on lifestyle physical activity (LIFEPA, min/day, 760–1951 cpm), time spent on moderate-to-vigorous intensity physical activity (MVPA time, min/day, 1952 ≥ cpm), time spent on health-enhancing physical activity (HEPA time, min/day) and steps per day [19]. HEPA was defined as continuous MVPA lasting at least 10 minutes at a time according to the current physical activity recommendation [1]. The validity and reliability of the Actigraph GTX3 has been shown to be similar to the GT1M devices in laboratory testing and for the measurement of everyday activities [20,21]. The ActiLife accelerometer software (ActiLife version 5) was used for data collection, and customised software was used for data reduction and analysis. In order to meet at least 80% of the data reliability criterion [18], at least three days of the seven days and for a minimum of 500 minutes per day is set as a minimum criterion for the representative data.

### Self-reported physical activity

Self-reported physical activity is measured with questions that evaluate participants’ physical activity level during the last seven days at moderate intensity and vigorous levels. Questions include all the activity for which the physical effort is moderate or harder, including transportation to work and leisure time physical activity [17]. During the accelerometer measurements participants will also keep a diary, which includes the type and duration of physical activity.

### Secondary outcome measures

#### Beliefs and intentions for exercise

Intentions and beliefs for physical activity are measured with the questionnaires, which measure adoption self-efficacy concerning physical activity with five items [22], barriers regarding exercise with five items [22], action planning for exercise with four items and coping planning for exercise with four items [23]. Response alternatives range from 1 (very certain I cannot) to 4 (very certain I can).

#### Psychological and depressive symptoms

Psychological symptoms are measured with the General Health Questionnaire (GHQ-12), which has been widely used to evaluate psychological distress in population-based studies. GHQ-12 is the shortened version of the GHQ-60 and GHQ-36, which has produced results comparable to the longer versions of the GHQ [24]. It contains 12 items describing psychological distress, responding to “how a subject feels about their present state over the past few weeks”. The scale has a four-point response scale, which was scored using a bimodal scale (0, 0, 1, 1) on the basis of the presence of symptoms: “not at all” (0), “same as usual” (0), “rather more than usual” (1), and “much more than usual” (1). The total score, obtained by summing the scores of the individual items, is a measure for severity of illness.

Psychological symptoms are also measured with the Symptom Checklist-90, SCL-90 [25,26]. Participants are asked to score the questionnaire’s 90 items using a five-point Likert scale, indicating the rate of occurrence of the symptoms during the time reference. The instrument’s global index of distress is the Global Severity Index (GSI), which is the mean value of all of the items.

Depressive symptoms are measured with the Beck Depression Inventory, BDI-II [27,28]. The BDI-II is a 21-item scale measuring depression symptoms, including components from cognitive, behavioural, affective, and somatic aspects. Based on the scores, depressive symptoms can be categorised into no/minimal depression (0–13 points), mild depression (14–19 points), moderate depression (20–28 points), and severe depression (29–63 points) levels.

#### Mindfulness skills

The ability to be in the present moment is measured using the Kentucky Inventory of Mindfulness Skills, KIMS. The KIMS is a 39-item self-report inventory used to assess mindfulness skills. The questionnaire contained four different specific subscales or skills: 1) observing, 2) describing, 3) acting with awareness, and 4) accepting without judgment [29]. *The observing* subscale involves observing, noticing, or attending to various stimuli, including internal (cognitions, bodily sensations) and external phenomena (sounds, smells). *The describing* subscale measures participant ability to describe, label, or notice observed phenomena by applying words in a non-judgmental way. The *acting with awareness* subscale measures ability to be attentive and engage fully in one’s current activity. The subscale of *accepting without judgment* measures how reality is allowed without judging, avoiding, changing, or escaping from it. Participants rated each item on a 5-point Likert type scale ranging from 1 (never or very rarely true) to 5 (almost always or always true). Items reflected either direct descriptions of the mindfulness skills or the absence of that skill, and were reverse scored [29].

#### Psychological flexibility

Psychological flexibility is assessed using the Acceptance and Action Questionnaire, AAQ-2 [6], which is a 10-item Likert-type questionnaire that assesses individuals’ ability to take a non-elaborative, non-judgmental approach to their internal events, so that they can focus on the present moment and act in a way that is congruent with their values and goals, rather than with their internal events (e.g., fears, urges, prejudices). The questions of the AAQ-2 are based on the following statements: 1) It’s okay if I remember something unpleasant, 2) My painful experiences and memories make it difficult for me to live a life that I would value, 3) I’m afraid of my feelings, 4) I worry about not being able to control my worries and feelings, 5) My painful memories prevent me from having a fulfilling life, 6) I am in control of my life, 7) Emotions cause problems in my life, 8) It seems that most people are handling their lives better than I am, 9) Worries get in the way of my success, and 10) My thoughts and feelings do not get in the way of how I want to live my life. The AAQ has been reported to have good reliability and validity [30,31].

### Statistical analysis

Data will be analyzed using the Mplus statistical package 7.1 [32]. The intervention effect on primary and secondary outcome measures will be estimated and tested for significance. Analyses will be controlled for possible baseline differences between the ACT + FB and FB groups. Cohen’s *d* will be used to estimate the effect size of the intervention effect. An effect size of 0.2 is considered small, 0.5 is medium, and 0.8 is large [33]. Furthermore, differences in the stability of the outcome measures between the ACT + FB and FB groups will be examined using multiple-group modeling techniques. Full information maximum likelihood (FIML) estimation under the assumption of data missing at random (MAR) will be used in analyzing incomplete data. As the normality assumption is violated, maximum likelihood with robust standard errors (MLR) will be used. The significance level of the study will be set at 0.05.

### Sample size

Power calculations were performed in order to determine the sufficient sample size to detect the significant intervention effect at a significance level of α = 0.05. Analysis was conducted for one of the primary outcome measures, objectively measured time spent in health-enhancing physical activity (HEPA time, min/day). A sample size of 100 (50 per group) would be sufficient to detect a medium-sized effect at the power level of 85%. In this case, a difference in a change in the HEPA time between the ACT + FB and FB groups equal to 5 minutes per day would be detected at appropriate levels of statistical power. Power calculations were based on Monte Carlo simulation studies and were performed by using the Mplus statistical package [32].

## Discussion

This article demonstrates the research protocol for a randomized controlled study focused on motivating physically more active lifestyles among physically inactive adults based on individuals’ own values. The study will assess the impact of intervention on physical activity levels and psychological well-being, and discuss the challenges and methodological problems in studying physically inactive participants.

Many authors have expressed the need for new theories and effective interventions to enhance physical activity [4]. To our knowledge, this is the first study focusing on motivating physically more active lifestyles by using value- and mindfulness-based approaches in combination with monitoring physical activity. This approach does not include traditional counseling or psycho-educational elements. Instead, the focus is on mindful behavioural change, and well-being and individual motivation based on one’s own values in life. Working with values and value clarification will provide ongoing direction and long term outcome goals in life. Instead, the actions are defined more as process goals, which can be set in the short term. Importance is also placed on the functions of the actions and the practices that work in order to achieve a meaningful life.

The study will provide novel information about new methods for motivating physically more active lifestyles and related challenges in conducting the study (e.g., recruiting physically inactive adults). In this study, the study population is limited to physically inactive adults. This is a very important starting point if the goal is to evaluate the workability of methods, which aims to motivate physically active lifestyles. The methods that work for physically more active adults may not be the best way to motivate least active ones. In addition, in practice it may be a challenge to find these physically inactive participants and get them involved. Methodologically it may also be demanding to keep the feedback group in the study, because they will receive the minor intervention. The study will provide more information of the psychological well-being of the physically inactive adults, which may be have an important role behind the physically active behaviour change. In addition, the qualitative and quantitative analysis of participants’ experiences will be of importance.

In conclusion, the study will explore the feasibility and efficacy of feedback and ACT based approaches in motivating physically more active lifestyles among physically inactive adults. The study will also examine the effectiveness on psychological well-being. The results will be discussed in the light of the ACT model and based on cognitive behavioural learning theories in order to bring new and alternative approaches to the field.

## Abbreviations

ACT: Acceptance and commitment therapy; FB: Feedback; CPM: Counts per minute; LPA time: Light intensity physical activity; LIFEPA: Lifestyle physical activity; MVPA: Moderate-to-vigorous intensity physical activity; HEPA: Health-enhancing physical activity; GHQ-12: General health questionnaire; SCL-90: Symptom Checklist-90; BDI-II: Beck depression inventory; KIMS: Kentucky inventory of mindfulness skills; AAQ-2: Acceptance and action questionnaire.

## Competing interests

The authors declare that they have no competing interests.

## Authors’ contributions

AMK is responsible for the research setting and contributed all the study phases in practice. She wrote the article. RL and THT have participated in, and significantly contributed to, the planning of the study design, coordination and hypothesis of the study. AK has been responsible for the statistical analysis and power calculations. JK has been part of the planning and carrying out practical implementation of the objective measurements including accelerometer, pedometer and body composition measurements. HH has been responsible for the accelerometer data. All authors read and approved the final manuscript.

## Pre-publication history

The pre-publication history for this paper can be accessed here:

http://www.biomedcentral.com/1471-2458/13/671/prepub
